# Supplementary material for: Compi: a framework for portable and reproducible pipelines
Source: PeerJ Comput Sci. 2021 Jun 18;7:e593. doi: 10.7717/peerj-cs.593 (PMC8237318; doi:10.7717/peerj-cs.593)
Supplement: Supplemental Information 1 [file peerj-cs-07-593-s001.docx]

**Supplementary File 1**. Command-line interface for the RNA-Seq Compi pipeline (<https://www.sing-group.org/compihub/explore/5d09fb2a1713f3002fde86e2>), obtained with the command compi run –p pipeline.xml --help.

usage: compi run <general-options> -- <pipeline-parameters>

where <general-options>: [-p <pipeline>] [-pa <params>] [-n <num-tasks>] [-l <logs>] [-lt <log-only-task>] [-nl <no-log-task>] [-st <single-task>] [-f <from>] [-a <after>] [-ut <until>] [-bt <before>] [-r <runners-config>] [-o] [-q] [-w] [-h]

--pipeline/-p

XML pipeline file (default: pipeline.xml)

--params/-pa

parameters file

--num-tasks/-n

maximum number of tasks that can be run in parallel. This is not equivalent to the number of threads the pipeline will use, because some tasks can be parallel processes themselves (default: 6)

--logs/-l

Directory to save tasks' output (stdout and stderr, in separated files). By default, no output is saved. If this option is provided, all task's output will be logged by default. You can select which tasks to log with --log-only-task or --no-log-task

--log-only-task/-lt

Log task(s). Task id(s) whose output will be logged, other tasks' output will be ignored. This parameter is incompatible with --no-log-task. If you use this option, you must provide a log directory with --logs. This option can be specified multiple times

--no-log-task/-nl

Do not log task(s). Task id(s) whose output will be ignored, other tasks' output will be saved. This parameter is incompatible with --log-only-task. If you use this option, you must provide a log directory with --logs. This option can be specified multiple times

--single-task/-st

runs a single task without its dependencies. This option is incompatible with --from, --after, --until and --before

--from/-f

from task(s). Runs the pipeline from the specific task(s) without running its/their dependencies. This option is incompatible with --single-task. This option can be specified multiple times

--after/-a

after task(s). Runs the pipeline from the specific task(s) without running neither it/them nor its/their dependencies. This option is incompatible with --single-task. This option can be specified multiple times

--until/-ut

runs until a task (inclusive) including its dependencies. This option is incompatible with --single-task and --before

--before/-bt

runs all tasks which are dependencies of a given task. This option is incompatible with --single-task and --until

--runners-config/-r

XML file configuring custom runners for tasks. See the Compi documentation for more details

--show-std-outs/-o

Forward task stdout/stderr to the compi stdout/stderr

--quiet/-q

Do not output compi logs to the console

--abort-if-warnings/-w

Abort pipeline run if there are warnings on pipeline validation

--help/-h

Show help of the specified pipeline

where <pipeline-parameters>: -ballgown_file <ballgown_file> -samples_class2_label <samples_class2_label> -samples_class1_label <samples_class1_label> -samples_dir <samples_dir> [-samtools <samtools>] -genome_index_dir <genome_index_dir> [-stringtie <stringtie>] -samples_stringtie_mergedannotation <samples_stringtie_mergedannotation> [-ballgown_script <ballgown_script>] -genome_index <genome_index> [-rscript <rscript>] -reference_annotation <reference_annotation> -samples_stringtie_mergelist <samples_stringtie_mergelist> -samples_alignment_dir <samples_alignment_dir> -genome_fasta <genome_fasta> -ballgown_dir <ballgown_dir> [-hisat2 <hisat2>] [-hisat2_index <hisat2_index>] -samples_stringtie_dir <samples_stringtie_dir>

samtools:

--samples_stringtie_mergelist/-samples_stringtie_mergelist

The path to the merge list file to feed StringTie with.

--stringtie/-stringtie

The executable of the StringTie command. (default: /opt/stringtie-1.3.1c.Linux_x86_64/stringtie)

--samtools/-samtools

The executable of the samtools command. (default: /opt/samtools-1.3.1/samtools)

--hisat2/-hisat2

The executable of the HISAT2 command. (default: /opt/hisat2-2.0.5/hisat2)

--samples_stringtie_dir/-samples_stringtie_dir

The directory where StringTie files should be placed.

--genome_index/-genome_index

The name for the genome index.

--reference_annotation/-reference_annotation

The path to the reference GTF file for the analysis.

--samples_alignment_dir/-samples_alignment_dir

The directory where aligned samples reads should be placed.

--samples_dir/-samples_dir

The directory containing the samples reads.

genome-index:

--genome_fasta/-genome_fasta

The reference genome.

--hisat2_index/-hisat2_index

The executable of the HISAT2 index command. (default: /opt/hisat2-2.0.5/hisat2-build)

--genome_index/-genome_index

The name for the genome index.

initialization:

--ballgown_file/-ballgown_file

The path to the Ballgown CSV file with the input data.

--ballgown_dir/-ballgown_dir

The directory where Ballgown files should be placed.

--samples_stringtie_mergelist/-samples_stringtie_mergelist

The path to the merge list file to feed StringTie with.

--samples_stringtie_dir/-samples_stringtie_dir

The directory where StringTie files should be placed.

--genome_index_dir/-genome_index_dir

The directory where genome indexes should be created.

--samples_alignment_dir/-samples_alignment_dir

The directory where aligned samples reads should be placed.

stringtie:

--samples_stringtie_mergelist/-samples_stringtie_mergelist

The path to the merge list file to feed StringTie with.

--stringtie/-stringtie

The executable of the StringTie command. (default: /opt/stringtie-1.3.1c.Linux_x86_64/stringtie)

--samtools/-samtools

The executable of the samtools command. (default: /opt/samtools-1.3.1/samtools)

--hisat2/-hisat2

The executable of the HISAT2 command. (default: /opt/hisat2-2.0.5/hisat2)

--samples_stringtie_dir/-samples_stringtie_dir

The directory where StringTie files should be placed.

--genome_index/-genome_index

The name for the genome index.

--reference_annotation/-reference_annotation

The path to the reference GTF file for the analysis.

--samples_alignment_dir/-samples_alignment_dir

The directory where aligned samples reads should be placed.

--samples_dir/-samples_dir

The directory containing the samples reads.

ballgown-class-1:

--ballgown_file/-ballgown_file

The path to the Ballgown CSV file with the input data.

--samples_stringtie_dir/-samples_stringtie_dir

The directory where StringTie files should be placed.

--samples_class1_label/-samples_class1_label

The label for the first class.

stringtie-merge:

--samples_stringtie_mergelist/-samples_stringtie_mergelist

The path to the merge list file to feed StringTie with.

--stringtie/-stringtie

The executable of the StringTie command. (default: /opt/stringtie-1.3.1c.Linux_x86_64/stringtie)

--reference_annotation/-reference_annotation

The path to the reference GTF file for the analysis.

--samples_stringtie_mergedannotation/-samples_stringtie_mergedannotation

The path to the merged annotation file created by StringTie.

ballgown-class-2:

--ballgown_file/-ballgown_file

The path to the Ballgown CSV file with the input data.

--samples_class2_label/-samples_class2_label

The label for the second class.

--samples_stringtie_dir/-samples_stringtie_dir

The directory where StringTie files should be placed.

ballgown-analysis:

--ballgown_dir/-ballgown_dir

The directory where Ballgown files should be placed.

--rscript/-rscript

The executable of the RScript command. (default: Rscript)

--ballgown_script/-ballgown_script

The path to the Ballgown DE script. (default: /opt/ballgown-differential-expression.R)

alignment:

--samples_stringtie_mergelist/-samples_stringtie_mergelist

The path to the merge list file to feed StringTie with.

--stringtie/-stringtie

The executable of the StringTie command. (default: /opt/stringtie-1.3.1c.Linux_x86_64/stringtie)

--samtools/-samtools

The executable of the samtools command. (default: /opt/samtools-1.3.1/samtools)

--hisat2/-hisat2

The executable of the HISAT2 command. (default: /opt/hisat2-2.0.5/hisat2)

--samples_stringtie_dir/-samples_stringtie_dir

The directory where StringTie files should be placed.

--genome_index/-genome_index

The name for the genome index.

--reference_annotation/-reference_annotation

The path to the reference GTF file for the analysis.

--samples_alignment_dir/-samples_alignment_dir

The directory where aligned samples reads should be placed.

--samples_dir/-samples_dir

The directory containing the samples reads.

stringtie-analysis:

--stringtie/-stringtie

The executable of the StringTie command. (default: /opt/stringtie-1.3.1c.Linux_x86_64/stringtie)

--samples_stringtie_dir/-samples_stringtie_dir

The directory where StringTie files should be placed.

--samples_alignment_dir/-samples_alignment_dir

The directory where aligned samples reads should be placed.

--samples_stringtie_mergedannotation/-samples_stringtie_mergedannotation

The path to the merged annotation file created by StringTie.
