# Supplementary material for: Compi: a framework for portable and reproducible pipelines
Source: PeerJ Comput Sci. 2021 Jun 18;7:e593. doi: 10.7717/peerj-cs.593 (PMC8237318; doi:10.7717/peerj-cs.593)
Supplement: Supplemental Information 2 [file peerj-cs-07-593-s002.docx]

**Supplementary File 2**. Implementation of a Nextflow example pipeline in Compi.

To illustrate the way of creating Compi pipelines and the main differences with other workflow management systems, we have implemented a Nextflow example pipeline in Compi. Specifically, we have chosen the BLAST example pipeline available here: https://github.com/nextflow-io/blast-example. The Compi pipeline is publicly available at Compi Hub (https://www.sing-group.org/compihub/explore/6087b19e6da27f0019a7db1b) and GitHub (https://github.com/sing-group/compi-blast-example). The complete code of both pipelines is also provided at the end of this file.

This pipeline is easy to understand and allows illustrating the main differences between both. The objective of this comparison is not to show the superiority of one tool over the other, but to allow figuring out in which scenarios Compi may be useful.

The pipeline definition in Compi starts with the version declaration and the pipeline parameters. As shown in the image below, each parameter have a short name (used for the command-line interface (CLI) generation), a description, and, optionally, a default value which is used in case the parameter is not specified by the user at runtime.


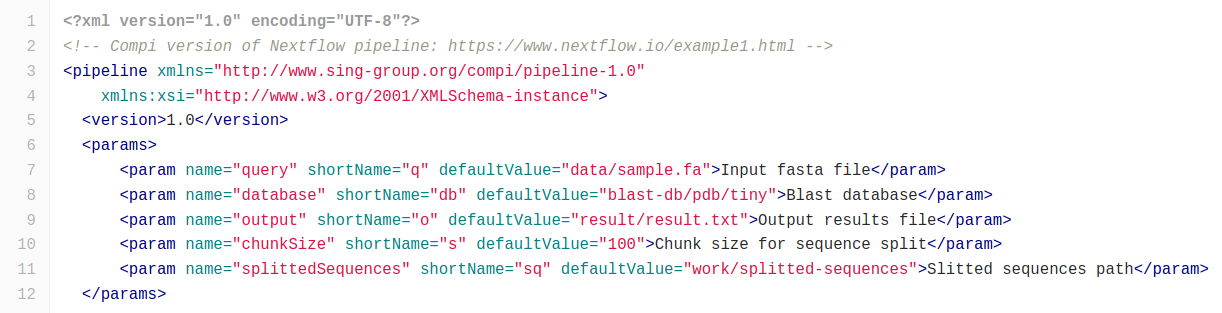


Similarly, the Nextflow pipeline also starts by declaring the pipeline input parameters with default values.


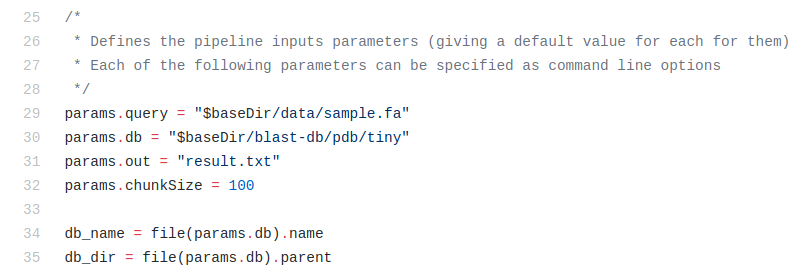


Then, the *“tasks”* block includes the definition of the different pipeline tasks. The Nextflow pipeline splits the input FASTA into chunks of the specified size and emits each block using a *“Channel”*. Whenever a new block is available in the channel, the *“blast”* process executes its code, which at the same time puts the outputs in another channel. In Compi, the first task, named *“splitFasta”*, splits the input FASTA file, saving each block of sequences in a different file under the directory specified by the *“splittedSequences”* parameter. Only when this task is finished, the next task (*“blast”*) starts to analyze each file. Since this task is a *“foreach”*, each file is analyzed in parallel (up to the maximum number of threads specified when running Compi).

After running BLAST, the next pipeline step is extracting the sequences associated to the BLAST hits using *“blastdbcmd”*. As before, the *“extract”* process of the Nextflow pipeline is executed each time a new value is available in the channel that connects this process with the previous *“blast”* process. The Compi pipeline does the same, since the *“extract”* foreach task is connected to the previous foreach at iteration level: they are binded foreachs (which is indicated by the “*” character in the *after=”*blast”*).


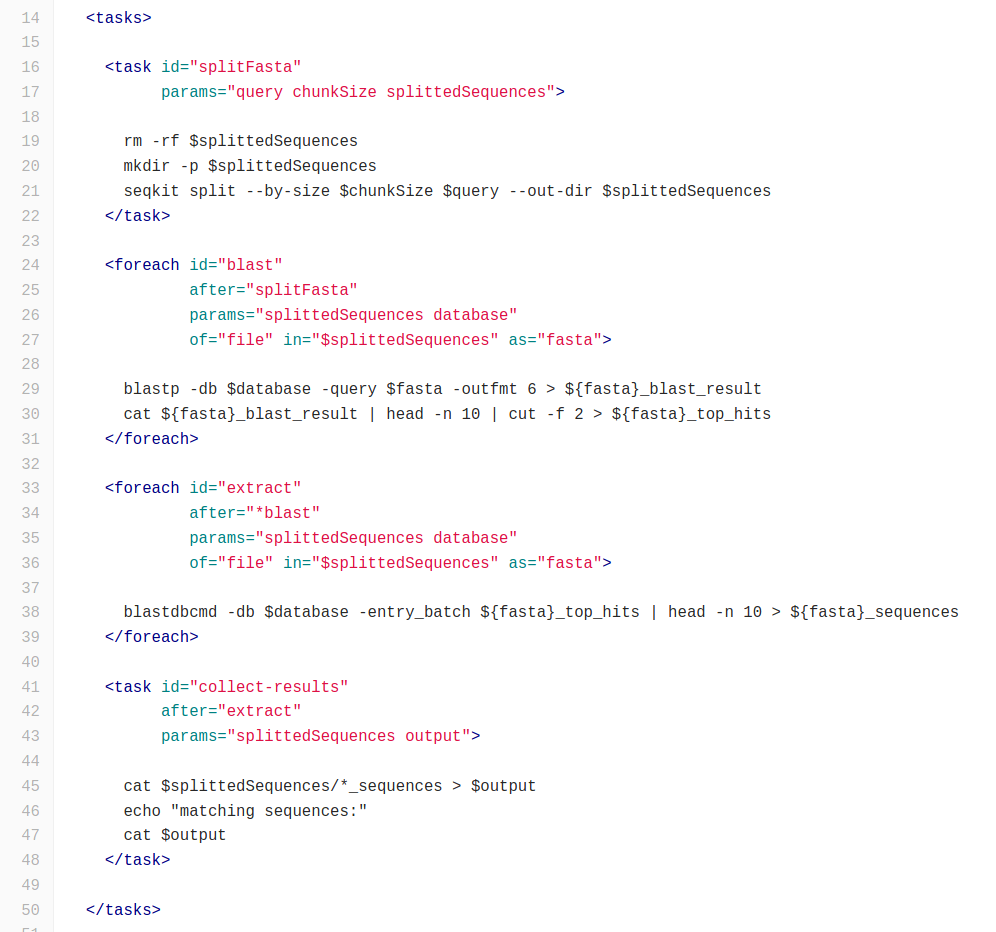


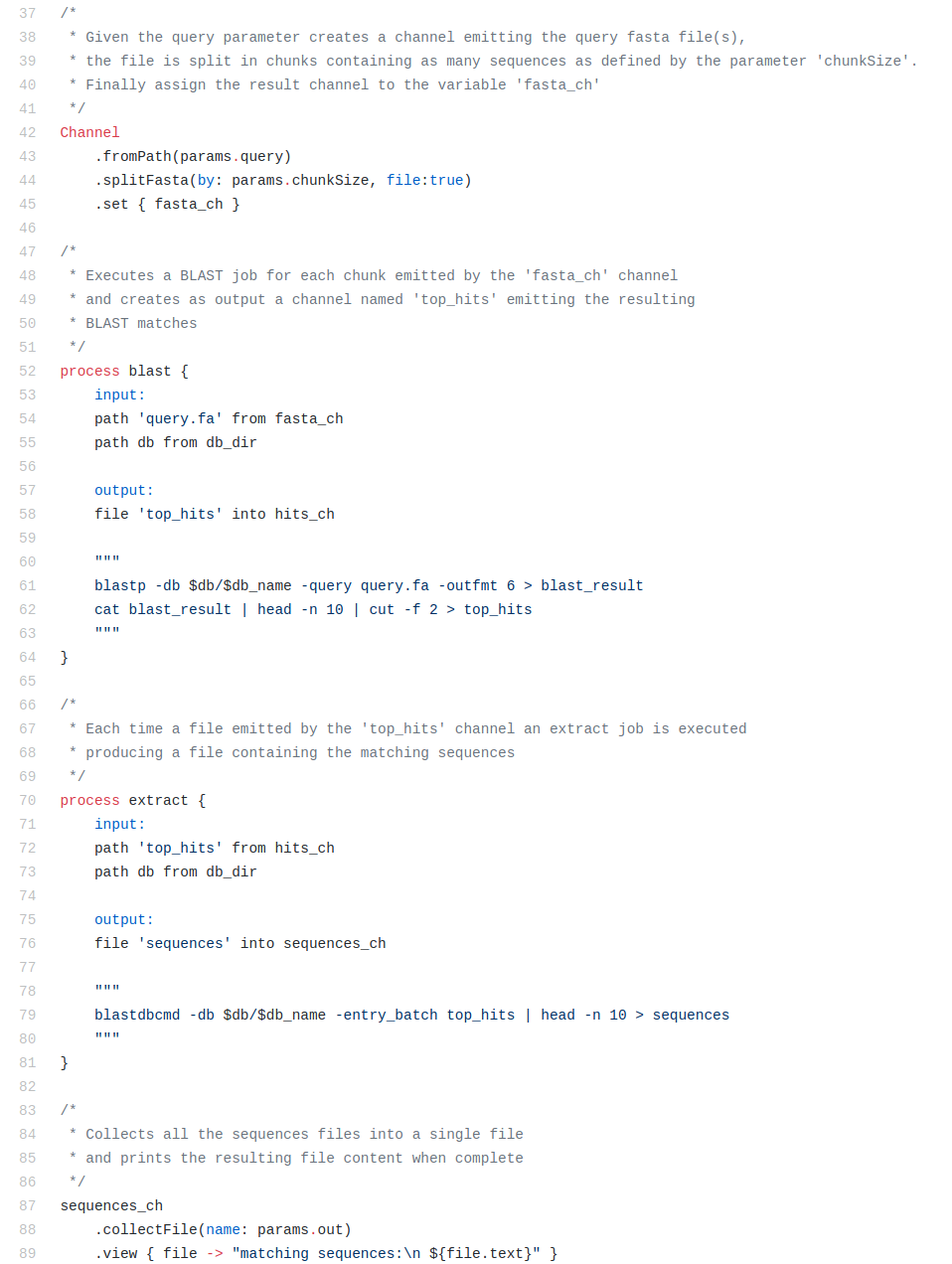


Finally, the Compi pipeline includes a *“metadata”* section that allows to describe the pipeline tasks in a human-readable manner. This provides a formal way of including the pipeline documentation, together with the parameter descriptions of the beginning, in the pipeline definition. Also, this information is used by the Compi Hub repository to automatically generate the online documentation.


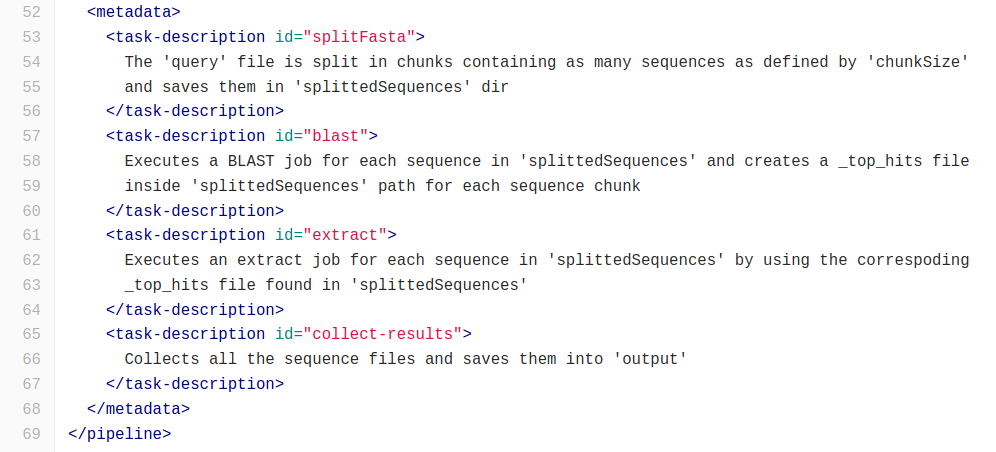


**Compi pipeline:** pipeline.xml file

<?**xml** version="1.0" encoding="UTF-8"?>

<!-- Compi version of Nextflow pipeline: https://www.nextflow.io/example1.html -->

<pipeline xmlns="http:**//**www.sing-group.org/compi/pipeline-1.0"

xmlns:xsi="http:**//**www.w3.org/2001/XMLSchema-instance">

<version>1.0</version>

<params>

<param name="query" shortName="q" defaultValue="data/sample.fa">Input fasta file</param>

<param name="database" shortName="db" defaultValue="blast-db/pdb/tiny">Blast database</param>

<param name="output" shortName="o" defaultValue="result/result.txt">Output results file</param>

<param name="chunkSize" shortName="s" defaultValue="100">Chunk size for sequence split</param>

<param name="splittedSequences" shortName="sq" defaultValue="work/splitted-sequences">Slitted sequences path</param>

</params>

<tasks>

<task id="splitFasta"

params="query chunkSize splittedSequences">

rm -rf $splittedSequences

mkdir -p $splittedSequences

seqkit split --by-size $chunkSize $query --out-dir $splittedSequences

</task>

<foreach id="blast"

after="splitFasta"

params="splittedSequences database"

of="file" in="$splittedSequences" as="fasta">

blastp -db $database -query $fasta -outfmt 6 > ${fasta}_blast_result

cat ${fasta}_blast_result | head -n 10 | cut -f 2 > ${fasta}_top_hits

</foreach>

<foreach id="extract"

after="*blast"

params="splittedSequences database"

of="file" in="$splittedSequences" as="fasta">

blastdbcmd -db $database -entry_batch ${fasta}_top_hits | head -n 10 > ${fasta}_sequences

</foreach>

<task id="collect-results"

after="extract"

params="splittedSequences output">

cat $splittedSequences/*_sequences > $output

echo "matching sequences:"

cat $output

</task>

</tasks>

<metadata>

<task-description id="splitFasta">

The 'query' file is split in chunks containing as many sequences as defined by 'chunkSize'

and saves them in 'splittedSequences' dir

</task-description>

<task-description id="blast">

Executes a BLAST job for each sequence in 'splittedSequences' and creates a _top_hits file

inside 'splittedSequences' path for each sequence chunk

</task-description>

<task-description id="extract">

Executes an extract job for each sequence in 'splittedSequences' by using the correspoding

_top_hits file found in 'splittedSequences'

</task-description>

<task-description id="collect-results">

Collects all the sequence files and saves them into 'output'

</task-description>

</metadata>

</pipeline>

**Nextflow pipeline:** pipeline.xml file

#!/usr/bin/env nextflow

/*

*

* Copyright (c) 2013-2018, Centre **for** Genomic Regulation (CRG).

* Copyright (c) 2013-2018, Paolo Di Tommaso and the respective authors.

*

* This **file** is part of 'Nextflow'.

*

* Nextflow is free software: you can redistribute it and/or modify

* it under the terms of the GNU General Public License as published by

* the Free Software Foundation, either version 3 of the License, or

* (at your option) any later version.

*

* Nextflow is distributed **in** the hope that it will be useful,

* but WITHOUT ANY WARRANTY; without even the implied warranty of

* MERCHANTABILITY or FITNESS **FOR** A PARTICULAR PURPOSE. See the

* GNU General Public License **for** more details.

*

* You should have received a **copy** of the GNU General Public License

* along with Nextflow. If not, see <http://www.gnu.org/licenses/>.

*/

/*

* Defines the pipeline inputs parameters (giving a **default** value **for** each **for** them)

* Each of the following parameters can be specified as command line options

*/

params.query = "$baseDir/data/sample.fa"

params.db = "$baseDir/blast-db/pdb/tiny"

params.**out** = "result.txt"

params.chunkSize = 100

db_name = file(params.db).name

db_dir = file(params.db).parent

/*

* Given the query parameter creates a channel emitting the query fasta **file**(s),

* the **file** is split **in** chunks containing as many sequences as defined by the parameter 'chunkSize'.

* Finally assign the result channel to the variable 'fasta_ch'

*/

Channel

.fromPath(params.query)

.splitFasta(by: params.chunkSize, **file**:true)

.**set** { fasta_ch }

/*

* Executes a BLAST job **for** each chunk emitted by the 'fasta_ch' channel

* and creates as output a channel named 'top_hits' emitting the resulting

* BLAST matches

*/

**process** blast {

input:

**path** 'query.fa' **from** fasta_ch

**path** db **from** db_dir

output:

**file** 'top_hits' into hits_ch

"""

blastp -db $db/$db_name -query query.fa -outfmt 6 > blast_result

cat blast_result | head -n 10 | cut -f 2 > top_hits

"""

}

/*

* Each **time** a **file** emitted by the 'top_hits' channel an **extract** job is executed

* producing a **file** containing the matching sequences

*/

**process** **extract** {

input:

**path** 'top_hits' **from** hits_ch

**path** db **from** db_dir

output:

**file** 'sequences' into sequences_ch

"""

blastdbcmd -db $db/$db_name -entry_batch top_hits | head -n 10 > sequences

"""

}

/*

* Collects **all** the sequences **files** into a single **file**

* and prints the resulting **file** content when complete

*/

sequences_ch

.collectFile(**name**: params.**out**)

.**view** { **file** -> "matching sequences:\n ${file.text}" }
